# Enzymatic saccharification of *Typha domingensis* biomass: optimization and structural analysis

**DOI:** 10.1186/s12896-025-01091-0

**Published:** 2025-12-25

**Authors:** Sumera Zaki, Hammad Afzal Kayani, Uroosa Ejaz, Mohammed Alorabi, Abdullah K. Alanazi, Sheeba Naz, Muhammad Sohail, Zainul Abideen

**Affiliations:** 1 Department of Biosciences, Faculty of Life Sciences, Shaheed Zulfikar Ali Bhutto Institute of Science and Technology (SZABIST) University, Karachi, 75600 Pakistan; 2 Department of Biotechnology, College of Sciences, Taif University, Taif, 21944 Saudi Arabia; 3 Department of Chemistry, College of Science, Taif University, Taif, 21944 Saudi Arabia; 4 Dow College of Biotechnology, Dow University of Health Sciences, Karachi, 74200 Pakistan; 5 Department of Microbiology, University of Karachi, Karachi, 75270 Pakistan; 6 Dr Muhammad Ajmal Khan Institute of Sustainable Halophyte Utilization, University of Karachi, Karachi, 75270 Pakistan; 7 Sharjah, Seed Bank and Herbarium, University of Al Dhaid, Sharjah, United Arab Emirates

**Keywords:** Cellulase, Fermentation, *Neobacillus sedimentimangrovi* UE25, Saccharification, *Typha domingensis*

## Abstract

Halophytic plants have been recognized as a promising source of lignocellulosic biomass for bioconversion into value added products. In this study, cellulosic content of wild biomass from *Typha domingensis* was saccharified by cellulase from a thermophilic bacterium, *Neobacillus sedimentimangrovi* UE25. Indeed, this is the first report describing the use of enzymatic saccharification of *T. domingensis* biomass. Initially, the *N. sedimentimangrovi* UE25 cellulase was produced in the medium containing wild biomass from the halophyte plant. The data revealed that 159.84 IU mL^− 1^ endoglucanase was obtained when *T. domingensis* biomass was used as a substrate. The result of saccharification showed that 172 mg g^− 1^ reducing sugars were obtained when cellulosic content of *T. domingensis* biomass was saccharified by cellulase. The effect of temperature, cellulase units and incubation time on saccharification was determined by using a statistical tool, Central Composite design. Under optimized conditions of 14.6 endoglucanase units per g of substrate, temperature 60 °C and reaction time 13.4 h, 610.65 mg g^− 1^ reducing sugars were obtained after the saccharification of cellulose from *T. domingensis* biomass. Moreover, Fourier Transform Infrared spectroscopy and Scanning Electron microscopy affirmed structural changes in the substrate. These findings demonstrate that biomass from halophyte such as *T. domingensis* can serve as an efficient substrate for thermostable cellulase production and saccharification, contributing to sustainable bioresource utilization in saline environments.

**Supplementary Information:**

The online version contains supplementary material available at 10.1186/s12896-025-01091-0.

## Graphical Abstract

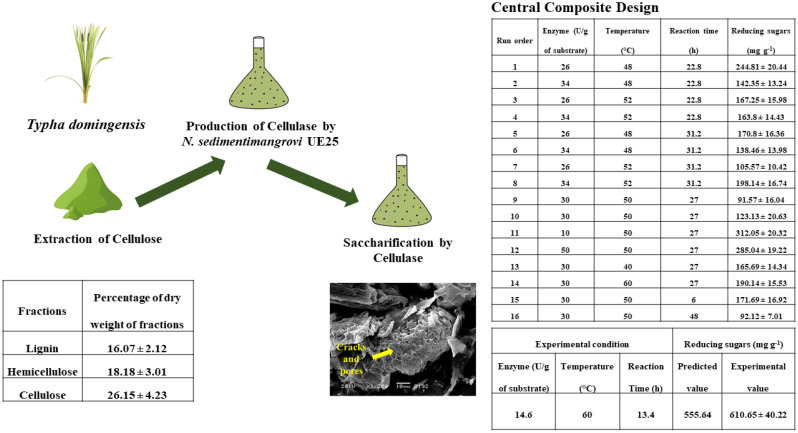

## Background

Halophytes grow in a broad range of saline environments such as in coastal areas, salt marshes, mudflats, inland deserts, and steppes [1]. Halophytes have developed a number of mechanisms which help them to grow and complete their life cycle in salinity-affected areas. These plants have evolved various mechanism for their long term survival in the saline environment including, presence of salt glands, compartmentalization, and salt exclusions at the root levels [2]. Although, in comparison to glycophytes, fewer halophytic genera have been reported, yet ~ 2,200 species of halophytes are found across the globe out of which 410 halophyte species inhabit Pakistan [3]. Reportedly 178 halophyte species are unique to Pakistan and have not been reported in any other region of the world [3]. These halophytic plants constitute 19% of the entire floral population of Pakistan.

Some native halophytic plants have been reported for their medicinal importance [4]. While some halophytes were reported for their high biomass ratio that can be converted into valuable products [5]. Like glycophytes, biomass from halophytic plants comprised of lignocellulose material, that is rich in three basic polymers, cellulose, hemicellulose and lignin. Cellulose accounts for up to 40% of plant biomass and consequently, the most abundant natural polymer on earth [6]. However, accessibility of cellulose for enzymatic hydrolysis is necessary for lignocellulosic biomass conversion into biofuel [7].

Among various biomasses from wild halophytes, *Typha domingensis* remained under-utilized. *T. domingensis* belongs to Typhaceae family, is a tall marshy grass that can reach a height of 2.0 to 2.5 m. This grass can be spotted flourishing both near the coast and inland areas typically flooded with sewage and rainwater [8]. It is a perennial grass, holds promise for bioenergy production and the remediation of heavy metals [9]. The potential of *T. domingensis* as a candidate for bioenergy and other applications has been highlighted previously [10].

Nonetheless, presence of lignin and crystalline nature of cellulose impede utilization of wild biomass from halophytes for conversion into biofuels and other bio-based products [11]. Hence, pretreatment of halophytic plant biomass before saccharification is crucial as it removes lignin and hemicelluloses, lowers the crystallinity of celluloses, and enhances the porosity of the materials which in turn facilitates the access of the saccharifying enzymes to the substrate [12]. Previous report showed that dilute acid pre-treatment of biomasses from different halophytic plants such as *P. antidotale*,* Phragmites karka*,* and H. mucronatum* improved enzymatic saccharification [13].

Among saccharifying enzymes, cellulase is the most important catalyst that converts cellulose into reducing sugar [14]. *Neobacillus sedimentimangrovi* UE25, a Gram-positive, endospore-forming, thermophilic bacterium has been reported as a source of thermostable [15] and end-product tolerant cellulase [15]. Saccharification mediated by thermostable cellulase prospects higher rates of reaction and high yield of oligosaccharides [16]. Yet, for efficient industrial process, factors affecting saccharification such as temperature, pH and reaction time need to be optimized to maximize the sugar yield [17]. In this context, statistical designs such as Central Composite design (CCD) has been used widely to obtain precise optimum level and to understand the interactive effects of the factors [18, 19].

In this context, this study holds the importance as it aimed to investigate biomass from salt tolerant halophytic plant (*T. domingensis*) for its potential to be utilized as a substrate for saccharification by using *N. sedimentimangrovi* UE25 cellulase. Moreover, factors affecting the saccharification were optimized at 5 levels using CCD. Structural changes in the halophytic plant were visualized using Scanning Electron microscopy and Fourier Transform Infrared spectroscopy.

## Materials and methods

### Collection of biomass from halophytic plant

Halophytic plant, *Typha domingensis* was collected from Dr. Muhammad Ajmal Khan Institute of Sustainable Halophytic Utilization, University of Karachi, Pakistan. The plant was identified by comparing with the already identified plant present at the Herbarium of University of Karachi, Pakistan. The collected plants were washed and air dried in the shade. Afterwards, the dried stem and leaves of plant were ground to the size of 250 µ.

### Extraction of cellulose from plant biomass

In order to extract the cellulose from *T. domingensis* biomass, different physical and chemical treatments were applied according to the method of Sridevi et al. [20] with slight modifications. Plant biomass (10 g) was added in 750 mL deionized water and boiled for 1 h. After filtration, the residues were dried at 60 °C until constant mass was obtained. After drying, each residue was carefully weighted. To extract hot-soluble ethanol fraction, 750 mL of ethanol was added to each of the residue left at the end of the hot water treatment and it was boiled for 1 h. The residue of each plant sample obtained was cooled and filtered. Residues were dried at 60 °C until constant mass was obtained and weighted. The difference between the solid residue, obtained at the end of the hot water treatment and the final residue after the ethanol process considered as hot-ethanol soluble fraction. To remove the lignin content, 300 mL of deionized water, 6 mL of sodium hypochlorite (NaOCl) and 20 mL of 10% (v/v) aqueous acetic acid solution were added to the residues, obtained at the end of ethanol process. Then the mixture was heated at 70 °C for 1 h. After that, acetic acid (20 mL) and sodium hypochlorite (6 mL) were added to the mixture and heated for 1 h. After cooling and filtration, the residues were dried at 60 °C until constant mass was obtained and then weighted. The difference in weight between residues obtained after ethanol treatment and the residues recovered after the present treatment considered as lignin content. To remove the hemicellulose, 200 mL of 24% KOH was poured to the solid residues obtained after lignin treatment and then allowed to settle at 20 °C for 2 h. Residues were then washed five times with deionized water, once with 5% aqueous acetic acid solution, once with deionized water then acetone and lastly with ether. Lastly, the sample was dried at 60 °C until constant mass was obtained and then the difference between the initial weight and that of the remaining residue after the removal of lignin was considered as the cellulosic content.

### Inoculum preparation

Cellulase producing bacterial strain, *N. sedimentimangrovi* UE25 (Genome Project ID JAJODE000000000.1) [21] was obtained from the Department of Microbiology, University of Karachi. To prepare inoculum, bacterial culture was grown in Nutrient broth and incubated for 24 h at 60 °C. Density of the inoculum was set to 0.3 OD_600nm_. Absorbance of the inoculum was recorded using UV/Vis Spectrophotometer, Beckman-Coulter, USA.

### Fermentation of extracted cellulose

To produce cellulase, mineral salt medium (MSM) [22] was used. Inoculum (10%) of bacterial culture was transferred in the fermentation medium containing 1% of extracted cellulose as a carbon source. The medium was incubated at 60 °C for 48 h. After incubation, the medium was centrifuged (Beckman Coulter, USA) at 2500 *x g* for 20 min. After centrifugation, cell free supernatant and pellet were separated. Cell free supernatant was used as a crude cellulase preparation and stored at -20 °C until used. The pellet (residual substrate) was dried in the incubator at 60 °C for 3 days and then kept in a cold and dry place.

### Enzyme assay

Enzyme assay was performed to determine the units of cellulase (endoglucanase) by using Dinitrosalicylic acid (DNS) method as described by Miller (1959). Carboxymethyl cellulose (CMC) was used as a substrate which was prepared by dissolving 0.1 g of CMC in 10 mL sodium-citrate buffer of pH 4.8. Crude cellulase (25 µL) was mixed with equal volume of substrate. The reaction mixture was incubated at 60 °C for 15 min. Afterwards, 150 µL of the DNS reagent was added to the reaction mixture and boiled for 5 min and then chilled on ice for 10 min. Deionized water (720 µL) was added and then the absorbance was measured at 540 nm. Before measuring the absorbance, spectrophotometer was calibrated using heat inactivated enzyme blank. Enzyme units were calculated using standard curve of glucose. One unit of the cellulase was defined as the amount of enzyme that is required to liberate 1 µmol of reducing sugar per min under standard assay conditions.

### Saccharification of extracted cellulose

The extracted cellulose for *T. domingensis* was used as a substrate for the saccharification experiment. Extracted cellulose (1 g) and sodium azide (0.2 g) were added in 100 mL sodium-citrate buffer (pH 4.8). Sodium azide was used to prevent bacterial contamination during saccharification experiment [24]. Crude cellulase (50 units) obtained from fermentation experiment was used for the saccharification. Reaction mixture was incubated at 60 °C for 24 h. After saccharification, mixture content was filtered to separate hydrolysate and biomass. The saccharified biomass was incubated at 60 °C for drying and then kept in the dry place. Reducing sugar content in the hydrolysate was measured.

### Reducing sugar assay

To estimate the amount of reducing sugars, DNS assay was [23] used. To 25 µL of each hydrolysate sample, 25 µL of sodium citrate buffer (PH 4.8) and 150 µL DNS reagent was added. Mixture was boiled for 5 min and then cooled on ice. Deionized water (720 µL) was added and absorbance at 540 nm was noted against blank. Blank was prepared by adding 25 µL sodium citrate buffer (pH.4.8) and 150 µL DNS reagent. After 5 min boiling, the mixture contents were cooled on ice cubes, followed by the addition of 720 µL deionized water. Absorbance was noted at 540 nm against a black which contained all of the above except for the sample. Standard curve of glucose was used to calculate the amount of reducing sugars in sample.

### Central composite design

In the current study, Central Composite design (CCD) was adopted to optimize the factors affecting the processes of saccharification of extracted cellulose from *T. domingensis* biomass. Three factors were investigated at five levels, including the cellulase units of *N. sedimentimangrovi* UE25 (10, 26, 30, 34 and 50 units), temperature (40, 48, 50, 52 and 60 °C) and reaction time (6, 27, 22.8, 31.2 and 48 h) (Table S1). Design was generated using Minitab software version 18. Total 16 experiment were performed to screen the effects of temperature, reaction time and enzymes units on saccharification processes. Hydrolysate obtained after experiments was assayed to measure the amount of glucose and reducing sugar by DNS method.

### Structural analysis of substrate

Fourier Transform Infrared spectroscopy (FTIR; JASCO FTIR-4200) and Scanning Electron microscope (SEM; JSM-6380 A, JEOL USA) were used to determine structural changes in extracted cellulose, and fermented, and saccharified substrate.

## Results and discussion

### Extraction of cellulose from *T. domingensis*

Cellulose was extracted from, *T. domingensis* biomass by sequential chemical extraction process. Cellulose is a valuable bioresource that can be used to manufacture value added products. Earlier, cellulose components extracted from halophytes including *Cressa cretica*,* P. karka*, and *Suaeda fruticosa* were used to make biocomposites [25]. In this study, the results showed *T. domingensis* biomass contained 16.07% lignin, 18.18% hemicellulose and 26.15% cellulosic content (Table 1). Earlier, Abideen et al. [26] reported that *T. domingensis* contained 4.67% lignin, 38.67% hemicellulose and 26.33% cellulose. Content of hemicellulose and lignin varied, however, cellulosic content was found to be ~ 26%. In an another study, it is also reported that *Typha* spp. (Cattails) contains 47.6% cellulose and 21.9% lignin [10]. The variation in chemical composition observed in *T. domingensis* compared to previously reported values in the literature may be attributed to several factors. Environmental conditions such as soil type, salinity, moisture availability, and nutrient levels can significantly influence the biosynthesis of lignocellulosic components in halophytic plants [27]. Additionally, the stage of plant maturity at the time of harvest plays a crucial role, as older plants tend to accumulate more lignin, while younger tissues may have higher cellulose and hemicellulose content [28, 29]. The extracted cellulose of *T. domingensis* was further used as a substrate for the production of thermostable cellulase and as a substrate for saccharification purpose. Ghazanfar et al. [30] also reported about the requirement of pretreatment of plant biomass to extract cellulose and to make the cellulosic content accessible for the microbial enzyme.

**Table 1:** Chemical composition of hemicelluloses lignin and cellulose in *Typha domingensis* plant biomass analyzed by gravimetric method

Fractions	Percentage of dry weight of fractions
	T. domingensis
Hot water-soluble fraction	39.1 ± 4.56
Hot ethanol-soluble fraction	0.5 ± 0.06
Lignin	16.07 ± 2.12
Hemicellulose	18.18 ± 3.01
Cellulose	26.15 ± 4.23

### Cellulase production under submerged fermentation

In this study, cellulase from *N. sedimentimangrovi* UE25 was obtained under submerged fermentation of the *T. domingensis* cellulose. Submerged fermentation is the most commonly used method for the large-scale production of cellulase and is particularly well-suited for microorganisms such as bacteria that require high moisture content for optimal growth [31]. The strain UE25 produced 159.84 IU mL^− 1^ endoglucanase in the medium containing extracted cellulose from *T. domingensis* biomass (Table 2). Previously, Ejaz et al. [32] also obtained higher enzyme yield (40.73 IU mL^− 1^) while using sugarcane bagasse-cellulose than the untreated sugarcane bagasse (22.72 IU mL^− 1^). While the same strain produced only 8.64 IU mL^− 1^ endoglucanase in the medium containing fungal-delignified sugarcane bagasse [33]. The results of this study indicate that *T. domingensis* biomass is a better substrate for cellulase production by *N. sedimentimangrovi* UE25 under submerged fermentation conditions. Its extracted cellulose resulted in enhanced enzyme yield (159.84 IU mL^− 1^). This highlights the potential of *T. domingensis* biomass as a sustainable and efficient feedstock for large-scale cellulase production.

**Table 2:** Production of cellulase enzyme by the fermentation of *Typha domingensis* using *Neobacillus sedimentimangrovi* UE25

Halophytic Plant	Cellulase
	(IU mL^− 1^)
*T. domingensis*	159.84 ± 13.02

### Saccharification of extracted cellulose from *T. domingensis*

Enzymatic saccharification has received more attraction recently because of being efficient and environmentally friendly [34]. In this study, enzymatic saccharification of extracted cellulose from biomass of *T. domingensis* was performed. Cellulose, a major structural component of plant biomass, is a linear polysaccharide composed of β-1,4-linked D-glucose units [35]. During saccharification, cellulase breaks down cellulose into oligosaccharides and glucose [36, 37]. The results of this study showed that 172 mg g^− 1^ reducing sugars were formed when cellulose from *T. domingensis* was saccharified (Table 3). Previously, Rashid et al. (2025) pretreated biomass of two halophytic plants, *Ipomoea pes-caprae* and *S. fruticosa* and obtained only 44 and 43 mg g⁻¹ reducing sugars, respectively. In another study, only 2.8 mg g⁻¹ reducing sugars were obtained after the saccharification of *Panicum antidotale* by cellulase of *Bacillus aestuarii* [13]. Whereas, saccharification of sugarcane bagasse yielded only 5 mg g^− 1^ reducing sugars after saccharification by cellulase from *Brevibacillus* sp [38]. Yet in another study, saccharification of alkali treated and untreated sugarcane bagasse by *Bacillus licheniformis* cellulase produced 0.3 and 0.69 mg g^− 1^ reducing sugars, respectively [39]. Among fungal strains, cellulase from *Cornu aspersum* [34] and *Aspergillus niger* [35] have been described for the saccharification of newspaper and rice straw, respectively. Nonetheless, the low yields necessitates optimization of the factors affecting the saccharification process [40].

**Table 3:** Saccharification of *Typha domingensis* by using crude cellulase of *Neobacillus sedimentimangrovi* UE25

Substrate	Reducing sugars (mg g^− 1^)
*T. domingensis*	172 ± 20.43

### Central composite design for the saccharification of *T. domingensis*-cellulose

Conditions affecting saccharification process were optimized through CCD as part of Response Surface methodology (RSM). This strategy has been adopted by various researchers, for instance, Ghazanfar et al. [41] used CCD to optimize the factors affecting saccharification of *Bombax ceiba*. In this study, amount of reducing sugars was taken as response that varied from 91.57 to 312.05 mg g⁻¹ in 16 experimental runs (Table 4). The experimental data from enzymatic saccharification were statistically investigated by the analysis of variance (ANOVA) (Table 5). The design was found to be significant as indicated by 0.002 *p* value and 97.45% regression square values. Temperature, cellulase units, and reaction time were critical factors that significantly influenced the efficiency of the enzymatic saccharification [42, 43]. Temperature affects both the activity and stability of the enzyme. As the temperature increases toward the enzyme’s optimum temperature, the kinetic energy of the molecules increases, enhancing enzyme-substrate interactions and accelerating the reaction rate [44]. However, temperatures beyond the optimal range can lead to enzyme denaturation, hindering catalytic activity and saccharification efficiency [45]. Likewise, higher enzyme loading generally increases the rate of cellulose hydrolysis by providing more active sites for substrate binding, but beyond a certain point, the effect plateaus due to substrate saturation or inhibition by the accumulated end-products [46, 47]. Reaction time is also one of the factors which affects enzyme-substrate interaction. Extended reaction times allow for complete hydrolysis of cellulose into fermentable sugars, but excessively long durations may not significantly enhance yield and could lead to enzyme degradation or increased process costs [48, 49]. Therefore, optimizing these parameters is essential to achieve maximum saccharification yield in a cost-effective and time-efficient manner.

**Table 4:** Central composite design for the saccharification of *Typha domingensis* by using crude cellulase of *Neobacillus sedimentimangrovi* UE25

Run order	Enzyme (U/g of substrate)	Temperature (°C)	Reaction time (h)	Reducing sugars (mg g^− 1^)
1	26	48	22.8	244.81 ± 20.44
2	34	48	22.8	142.35 ± 13.24
3	26	52	22.8	167.25 ± 15.98
4	34	52	22.8	163.8 ± 14.43
5	26	48	31.2	170.8 ± 16.36
6	34	48	31.2	138.46 ± 13.98
7	26	52	31.2	105.57 ± 10.42
8	34	52	31.2	198.14 ± 16.74
9	30	50	27	91.57 ± 16.04
10	30	50	27	123.13 ± 20.63
11	10	50	27	312.05 ± 20.32
12	50	50	27	285.04 ± 19.22
13	30	40	27	165.69 ± 14.34
14	30	60	27	190.14 ± 15.53
15	30	50	6	171.69 ± 16.92
16	30	50	48	92.12 ± 7.01

**Table 5:** Analysis of variance (ANOVA) of the model for the optimization of different factors that influence the saccharification process of *Typha domingensis*

Source	*P*-Value	F-Value
Model	0.002	19.12
Blocks	0.013	14.28
Linear	0.05	5.4
Enzyme units	0.234	1.83
Temperature	0.669	0.21
Reaction time	0.013	14.15
Square	0.001	38.06
Enzyme units*Enzyme units	0.004	25.63
Temperature*Temperature	0.01	16.19
Reaction time*Reaction time	0.015	13.16
2-Way Interaction	0.013	10.85
Enzyme units*Temperature	0.006	20.33
Enzyme units*Reaction time	0.02	11.19
Temperature*Reaction time	0.355	1.04
Lack-of-Fit	0.761	0.52

**Fig. 1:**
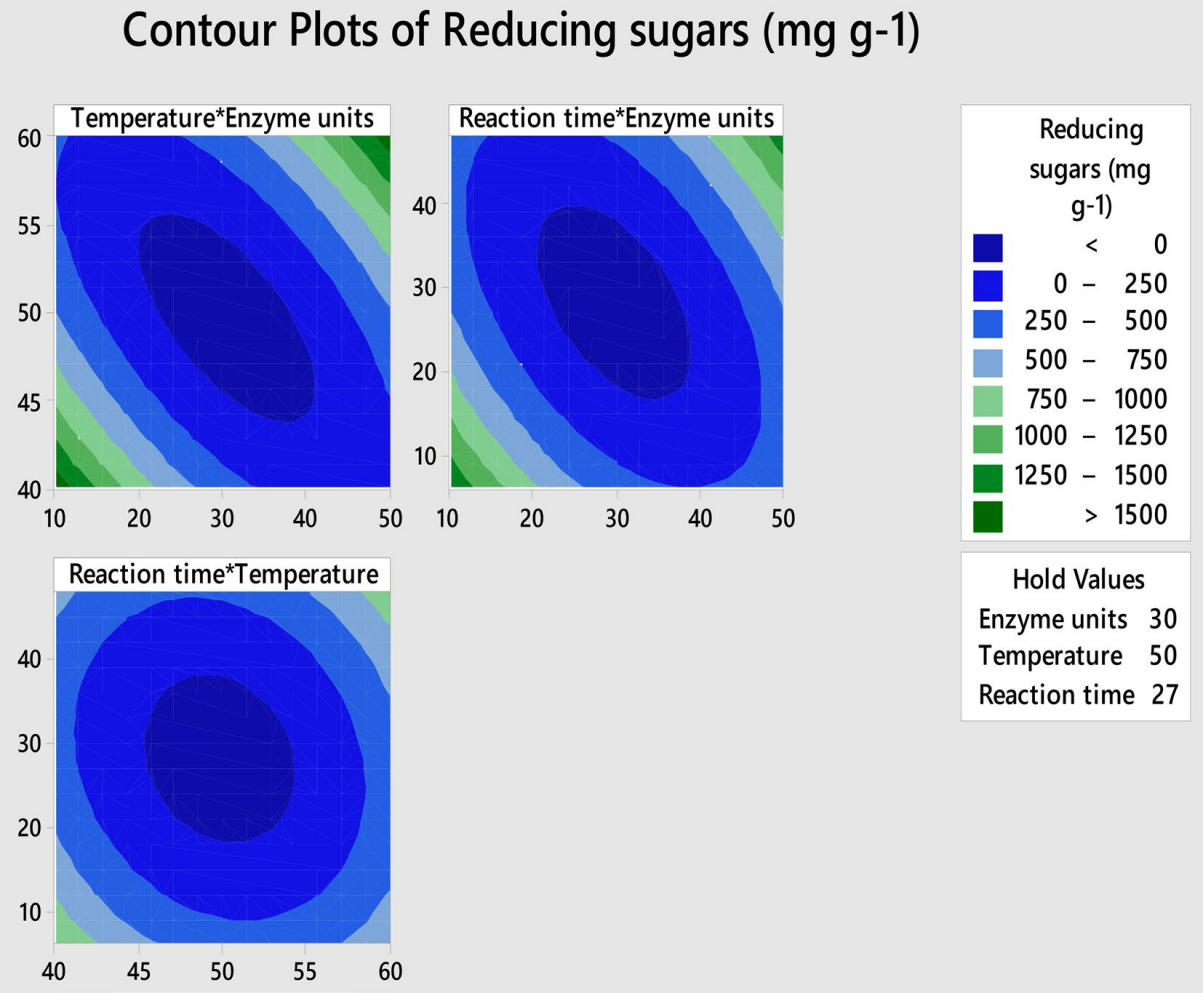
Contour plots showing the interaction between two factors and its effects on the process of saccharification

In this study, pareto chart and ANOVA showed that reaction time is the significant factor. Previously, Khan et al. [38] also reported reaction time as a significant factor affecting the saccharification of sugarcane bagasse by crude cellulase of *Brevibacillus* sp. MT5. Moreover, two-way interaction of enzyme units and reaction time significantly affected the saccharification process (Fig. S1) (Table 5). Normal probability chart also showed that all the experiments were closely related to the fit value and followed normal distribution (Fig. S2). Interaction of factors were studied through generating contour plots (Fig. 1). It was observed that interaction of temperature and cellulase units are directly proportional to each other (Fig. 1a). Similar results were observed for reaction time and enzyme units (Fig. 1b), where extended incubation in the presence of more enzyme promoted higher cellulose hydrolysis. Likewise, the interaction between reaction time and temperature was found to be directly proportional (Fig. 1c), implying that prolonged reaction duration and increased temperature collectively favor the saccharification process.

Furthermore, the CCD suggested response optimization experiment including the conditions of 14.6 U/g of substrate of cellulase units, 60 °C temperature and 13.4 h of reaction time to achieve maximum saccharification. The response optimization result for the saccharification of *T. domingensis*-cellulose was compared (Table 6) with the predicted value by software and it was found that experimental value (610.65 mg g^− 1^) was closely related to the predicted response (555.64 mg g^− 1^). After optimization, reducing sugar yield was increased by 3.55-fold. This strong agreement between predicted and experimental outcomes not only validated the robustness and reliability of the statistical model used but also highlighted the effectiveness of using RSM in bioprocess optimization. The remarkable improvement in saccharification efficiency suggests that *T. domingensis* possesses favorable structural and compositional characteristics making it an excellent candidate for the enzymatic hydrolysis.

**Table 6:** Response optimization for the saccharification of *Typha domingensis* showing positive correlation between experimental and predicted values

Experimental condition			Reducing sugars (mg g^− 1^)	
**Enzyme (U/g of substrate)**	**Temperature (°C)**	**Reaction Time (h)**	**Predicted value**	**Experimental value**
14.6	60	13.4	555.64	610.65 ± 40.22

### Structural analysis of *T. domingensis*-cellulose

**Fig. 2:**
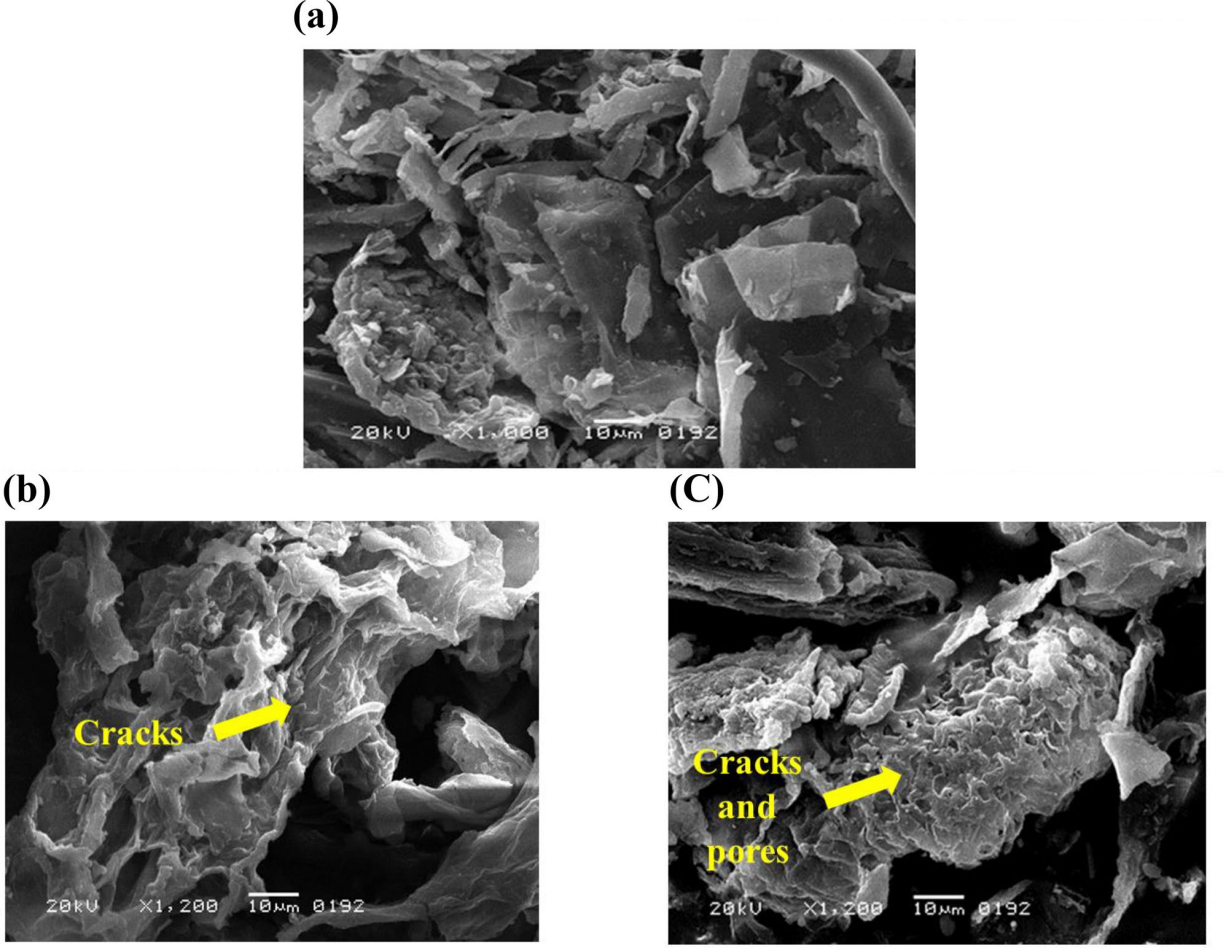
Scanning electron microscopy of (**a**) native (**b**) fermented and (**c**) saccharified *Typha domingensis*

Scanning electron microscopy of the native, fermented and saccharified cellulose of *T. domingensis* showed that in native substrate the structure was compact, well organized with tightly packed fibers (Fig. 2a). Whereas, fermented substrate revealed irregular and rough surface indicating bacterial action on the substrate (Fig. 2b). Similar changes were reported by Hassan et al. (2023) for the fermented substrate of *C. cretica* biomass where the submerged fermentation greatly altered the structure of cell wall by breaking bundle fiber due to which matrix was loosened and became fragile. Saccharified substrate displayed significant disruption of the cellulose fibers, with many broken and fragmented pieces. The surface was highly porous and irregular, indicating substantial hydrolytic degradation during saccharification process. Ansari et al. [13] also reported the similar morphology in the structure of *P. karka* after the saccharification process.

**Fig. 3:**
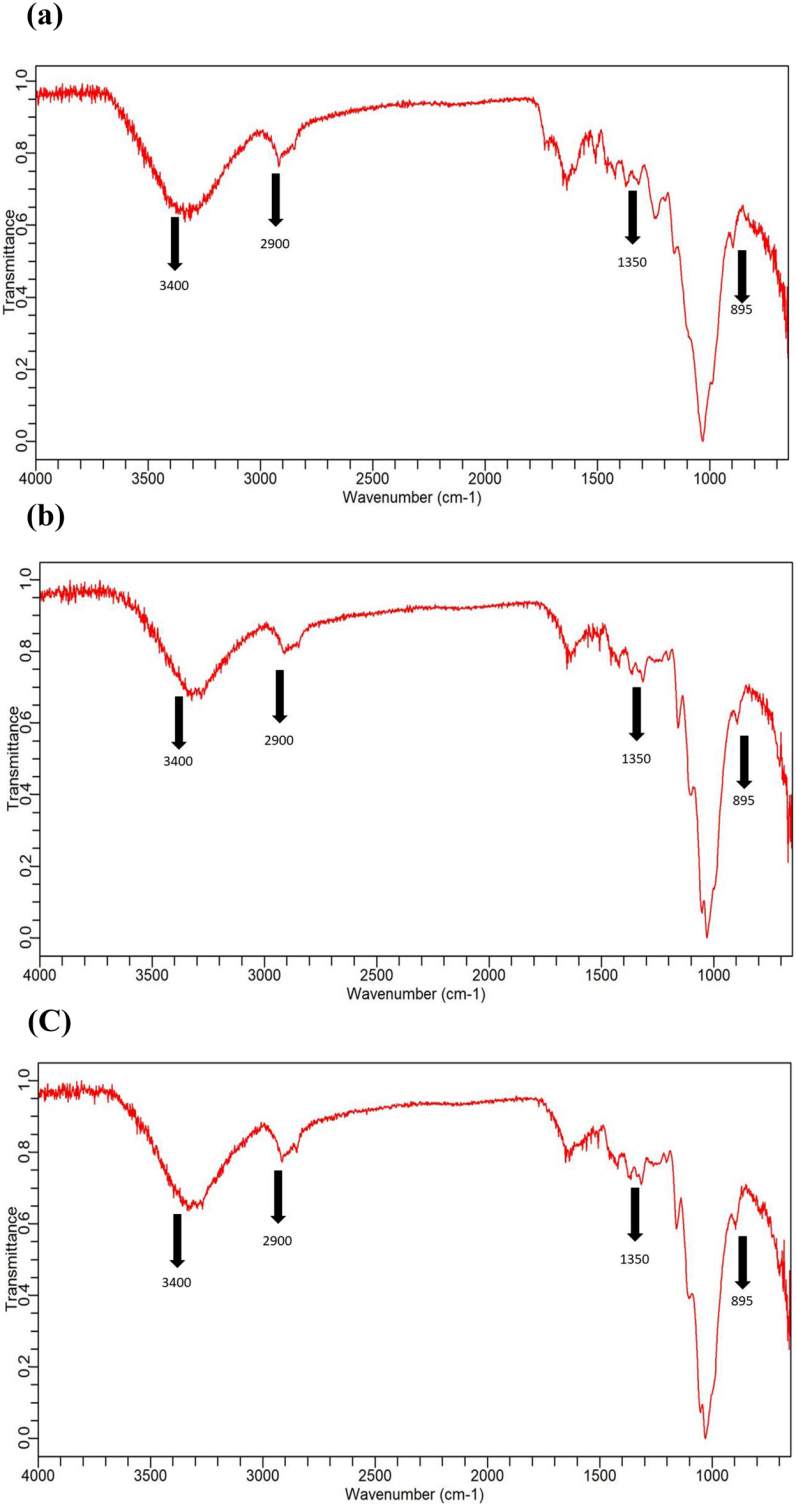
FTIR profiling of (**a**) native (**b**) fermented and (**c**) saccharified *Typha domingensis*

Changes in the functional group of the native, fermented and saccharified substrate was studied by FTIR that endorsed the effect of bacteria and its enzyme on the plant biomass component (Fig. 3). FTIR can detect changes in absorption band and peaks pattern reflecting changes in the biomass structure [13]. All the samples were scanned at wave lengths from 650 to 4000 cm^− 1^ and distinctive absorption bands of polysaccharide in native plant samples were evident. The bands in the region of 3000 of 3400 cm^− 1^ were because of the presence of hydroxyl group [50]. The broad-band region around 2900 cm^− 1^ was of C–H stretching vibration which is part of cellulosic component [51]. The change in the peaks around 1350 cm^− 1^ is ascribed to the CH group in a glucose unit, whereas, the band at 895 cm^− 1^ showed the linkage of β-glycosidic [52]. Furthermore, the stretching vibration of C–O–C of glycosidic structure were revealed by distinct band around 1000 cm^− 1^ [53]. Conversion of cellulosic component into reducing sugars and other components was observed as a result of bacterial enzyme action, evidenced by changes in cellulosic peaks.

## Conclusion

In this study, potential of biomass from a halophytic plant (*T. domingensis*) as a source of cellulose and its subsequent saccharification was evaluated by utilizing cellulase from the thermophilic bacterial strain, *N. sedimentimangrovi* UE25. Extracted cellulose was used as a fermentation substrate and also for saccharification processes. It was found that bacterial strain, *N. sedimentimangrovi* UE25 produced 159.84 IU mL^− 1^ cellulase by fermenting *T. domingensis*-cellulose. Optimized conditions for saccharification as predicted by Central Composite design included 14.6 U of cellulase per g of substrate, 60 °C temperature and 13.4 h reaction time; a process under optimized conditions yielded 610.65 mg g^− 1^ reducing sugars. Results of SEM and FTIR analysis showed changes in surface morphology and structure of native, fermented and saccharified substrate. Therefore, this study concludes that cellulose extracted from the halophytic plant biomass has potential to be tapped as sustainable substrate for both fermentation and enzymatic saccharification processes and the released reducing sugars can be subsequently used for different value-added product such as, prebiotics. However, purification of the oligosaccharides and further investigations are necessary to advance this area of research. Moreover, future studies exploring other microbial strains to obtain more enzyme titers and reducing sugars yield will strengthen the halophyte biomass based biorefineries.

## Supplementary Information

Below is the link to the electronic supplementary material.

Supplementary Material 1

## Data Availability

The data associated with this manuscript has been provided in a supplementary file available online.
