# Supplementary material for: Enzymatic saccharification of Typha domingensis biomass: optimization and structural analysis
Source: BMC Biotechnol. 2025 Dec 25;26:10. doi: 10.1186/s12896-025-01091-0 (PMC12849741; doi:10.1186/s12896-025-01091-0)
Supplement: Supplementary file 1 — Supplementary Material 1 [file 12896_2025_1091_MOESM1_ESM.docx]

# **Enzymatic saccharification of *Typha domingensis* biomass: Optimization and structural analysis**

Sumera Zaki^a^, Hammad Afzal Kayani^a*^, Uroosa Ejaz^a*^, Mohammed Alorabi^b^, Abdullah K. Alanazi^c^, Sheeba Naz^d^, Muhammad Sohail^e^_,_ Zainul Abideen^f^

^a^Department of Biosciences, Faculty of Life Sciences, Shaheed Zulfikar Ali Bhutto Institute of Science and Technology (SZABIST) University, Karachi 75600, Pakistan

^b^Department of Biotechnology, College of Sciences, Taif University, 21944 Taif, Saudi Arabia

^c^Department of Chemistry, College of Science, Taif University, 21944 Taif, Saudi Arabia

^d^Dow College of Biotechnology, Dow University of Health Sciences, Karachi-74200, Pakistan

^e^Department of Microbiology, University of Karachi, Karachi-75270, Pakistan

^f^Dr Muhammad Ajmal Khan Institute of Sustainable Halophyte Utilization, University of Karachi, Karachi-75270, Pakistan

*Corresponding author: [uroosa.ejaz@szabist.edu.pk](mailto:uroosa.ejaz@szabist.edu.pk) ORCID: 0000-0001-5595-1370; [hammad.afzal@szabsit.edu.pk](mailto:hammad.afzal@szabsit.edu.pk) ORCID: [0000-0002-3258-0535](https://orcid.org/0000-0002-3258-0535)

**Supplementary File**

**Fig. S1** Pareto chart showing the effects of factors on the process of saccharification

**Fig. S2** Normal probability plot showing the normal distribution of the saccharification data

**Table S1** Factors and their levels employed to screen the effect of temperature, reaction time and enzymes units on scarification processes

| **Factors** | Level of factors | | | | |
| --- | --- | --- | --- | --- | --- |
|  | 1 | 2 | 3 | 4 | 5 |
| Enzyme units (U/g of substrate) | 10 | 26 | 30 | 34 | 50 |
| Temperature (°C) | 40 | 48 | 50 | 52 | 60 |
| Reaction time (h) | 6 | 22.8 | 27 | 31.2 | 48 |
